# Across preclinical and clinical platforms, approved and investigational psychiatric drugs share pathways and associate with similar molecular functions

**DOI:** 10.3389/fddsv.2025.1686789

**Published:** 2026-03-03

**Authors:** Emily Su, Nico Matthew S. Valencia, Jarod Le, Emily Liu, Jennifer L. Wilson

**Affiliations:** 1 Computational and Systems Biology, University of California Los Angeles, Los Angeles, CA, United States; 2 College of Natural and Applied Sciences, Division of Natural Science, Biology Program, University of Guam, Mangilao, Guam; 3 Harvard Kenneth C. Griffin Graduate School of Arts and Sciences, Research Scholar Initiative, Cambridge, MA, United States; 4 Department of Neuroscience, Virginia Tech, Blacksburg, VA, United States; 5 Division of Clinical Trials and Biostatistics, Mayo Clinic, Rochester, MN, United States; 6 Department of Bioengineering, University of California Los Angeles, Los Angeles, CA, United States

**Keywords:** drug modeling, drug screening, network pharmacology, systems biology, translational psychiatry

## Abstract

**Introduction:**

Translating findings from cell and animal models to the clinic is difficult because these systems insufficiently capture human disease biology. Computational approaches, notably pathways modeling, have overcome interspecies differences by mapping shared signaling pathways between species. We considered whether this approach could be generalized to bridge animal and virtual drug screening data to effects measured in patients. We emphasized translational psychiatry datasets because of the unmet need for effective therapies.

**Methods:**

We conducted three parallel analyses using published drug screening data in zebrafish, structure-function computational screening data, and clinically-reported efficacy data for experimental and approved antipsychotic drugs and predicted drug pathways using the PathFX algorithm

**Results:**

Despite choosing screens developed for unique aspects of psychiatry and without careful curation of shared drugs, we found pathways associated with drugs that performed “well” across these distinct platforms—specifically, among drugs measured as favorable (expected to improve disease) or unfavorable (expected to aggravate disease) across screens, these drugs connected to distinct pathway proteins; however, these drugs are connected to similar protein families and shared Gene Ontology functional terms. By comparing screens, we discovered that favorable drugs may influence G-proteins, solute carrier family proteins, adrenoceptors, and steroid hydroxylase activity and that unfavorable drugs affect serotonin receptors and phosphodiesterase activity.

**Discussion:**

This suggests that predictive platforms could emphasize functional information as features that could overcome differences in distinct screening platforms to eventually improve translational approaches.

## Introduction

The translation of preclinical models to patients is notoriously difficult because model systems (e.g., animal and cellular models) insufficiently capture human disease biology (Plenge et al., 2013). In psychiatry, many diseases lack effective treatments, and the translation of novel therapies can be challenging. While there are several animal behavioral models, there are few consensus assays for early lead development (Licinio, 2011; Pike et al., 2021), and information can be lost when translated across these platforms (Fulford et al., 2014). One of the remaining translational challenges is the extent to which *in vitro* or *in vivo* models yield clinically successful candidates.

Novel computational approaches can offer insights if they extend beyond direct one-to-one mapping between model systems and patients. As a means of better bridging animals and humans, Brubaker et al. (2019) discovered that a semi-supervised neural network successfully inferred human biology from mouse transcriptomics for 36 case study phenotypes. While mouse models did not directly predict differentially expressed genes in human samples, the semi-supervised models identified signaling components that were represented in human samples. This suggested that computational models of signaling pathways may be able to “smooth” out the differences across different model systems. Ruiz et al. (2021) also incorporated Gene Ontology (GO) functional information with protein interaction networks to better predict drug effects. This suggests that the analysis of pathway-level effects may lead to better modeling of drug-induced effects. We considered that pathways modeling could have utility for identifying shared effects in psychiatric drug development pipelines.

Network medicine is one way to model the potential pathway effects of drugs, but the methods are underutilized in industry due to their tendency to overpredict drug effects (Harpaz et al., 2017; Wilson et al., 2022a; Alidoost and Wilson, 2024). We originally developed the PathFX algorithm to identify signaling pathways possibly perturbed by drugs and to predict drug phenotypic effects (Wilson et al., 2018). Like Ruiz et al. (2021) and Guney et al. (2016), our method identifies connections between drug targets and intend-to-treat disease genes using protein–protein interactions (PPIs). Algorithms like PathFX include high-quality interactions between drug target proteins and downstream proteins using mathematical formalisms that prioritize local, high-scoring interactions, comparing these selections to scores distributed throughout the network. Scores that reflect the likelihood of an interaction facilitate a way to broadly search the interaction network, but identified pathways require dedicated experiments to confirm predicted pathways. Advantageously, algorithms like PathFX yield interpretable predictions as they “connect the dots” from a drug target to verified (e.g., an intend-to-treat disease or clinically observed side effect) and predicted drug effects; sometimes a disease or side effect is directly associated with a drug target and sometimes through a downstream protein. Although these predicted pathways lack systematic validation, we and others have successfully used them to predict drug effects and extended them to more complex effects, including drug–drug interactions (Zitnik et al., 2018; Yoo et al., 2018; Wilson et al., 2022b). We observed that verified connections were shared with other, unvalidated predicted drug effects. In previous research, we found that comparing network-predicted effects with phenotypic drug screens supported network predictions as possible repurposing candidates, specifically in psychiatry (Bowen et al., 2023). We had used a phenotypic screen for drugs that reduced microglial phagocytosis, hypothesizing that a reduction in this process could mitigate symptoms associated with schizophrenia, and produce favorable outcomes. We discovered that pathways that model supported drug effects observed in microglia—specifically where drug predictions were connected through the beta-2 adrenergic receptor, ADRB2—were often associated with strong effects in the phenotypic screen. This analysis provided the rationale for using predicted pathways to describe drug effects and specifically emphasized the utility of psychiatry-associated predictions.

In this study, we wanted to understand the generalizability of identifying core signaling components across translational systems and bridging preclinical effects to clinical data. We first predicted pathways for drug candidates prioritized from computational and *in vivo* screens and compared these to pathway predictions for antipsychotic drugs used in the clinic. We hypothesized that data from multiple drug screens could be used to find proteins that have the most impact in the PPI network and that discovering these shared proteins could improve the identification of new treatments. Because we picked a diversity of screens, we initially hypothesized that pathways analysis of drugs prioritized by an *in silico* screen or one conducted in zebrafish would yield little overlap. Instead, we thought this comparison may set a “floor” for the interchangeability of these tools. However, we discovered that these screens enriched for drugs with pathway connections to similar protein families and GO biological processes. This study provides a strong rationale for embedding pathway-based drug effect prediction throughout predictive, translational pipelines in psychiatry.

## Methods

### Acquiring and parsing screening data: *in silico* screen

For the computational screen, we used SWEETLEAD validation data from Gravina et al. (2022) and selected drugs predicted to increase or decrease phagocytosis through their optimal machine learning approach. For context, this machine learning algorithm was built to predict novel compounds that could prevent microglial phagocytosis using chemical structure as input. The algorithm was trained on another published dataset from Bowen et al. (2023) in which they treated microglia with several compounds and experimentally measured phagocytosis in cells. The later prediction on the untested SWEETLEAD data prioritized several compounds with predicted effects on phagocytosis that were computationally, but not experimentally, validated.

We accessed the data directly from the first author, and although we cannot directly release the data, we provide code for parsing the data in our GitHub. The data were organized by chemical active ingredient name, drug dose, and predicted effect (i.e., “increase phagocytosis,” “decrease phagocytosis,” or “no effect”). In total, 306 drugs were predicted to increase phagocytosis and 627 drugs predicted to decrease phagocytosis at least one dose (for more details about their methods, see Gravina et al., 2022).

Then, we converted the drug names into DrugBank IDs using string matching between the published data and a mapping file from DrugBank version 5.1.6 which included active ingredient names and DrugBank identifiers. String matching prioritized compounds with similar active ingredient names in both datasets (e.g., “Ketamine” or “Verapamil”). Compound names or different formulations were skipped (e.g., “Verapamil Hcl” or “Verapamil Hydrochloride” contained in the SWEETLEAD data) because these were not provided as common names in DrugBank. Future efforts could consider methods for synchronizing results between different formulations. The name conversion code is provided in the study GitHub (see Data Availability). We were able to convert 285 (30.5%) of the original drugs (81 which were predicted to increase phagocytosis and 204 predicted to decrease) in the validation dataset and analyze them in PathFX. These DrugBank IDs are also included in Supplementary File S2.

### Acquiring and parsing screening data: zebrafish phenoscores

For the *in vivo* screen, we downloaded phenoscore data from McCarroll et al. (2019) from their Zenodo page (https://zenodo.org/records/3336616#.YrzrrRPMLLA). For context, this screen was developed to test compounds that could elicit “paradoxical excitation,” a phenomenon where anesthetics can also increase brain activity, in zebrafish and was designed to inform general central nervous system drug discovery. We specifically acquired file Figure_2b.csv, which contained chemical names and “1-corr” values which represented the drugs’ phenoscores. We capitalized chemical names provided in Figure_2b.csv and retained drugs with direct name matches in the DrugBank vocabulary. As before, we skipped different formulations that were not direct matches to DrugBank names or synonyms (e.g., “Riluzole hydrochloride” or “Arecoline hydrobromide”). For drugs tested multiple times, we retained the max reported phenoscore. We found 459 chemical names with direct DrugBank IDs, of which 359 had documented protein-binding targets and a final set of 327 for which PathFX made predictions (i.e., these drugs had targets that were in the PathFX interactome). Again, we provide our code for this process in GitHub.

### Acquiring and parsing screening data: clinical meta-analysis

Finally, for the clinical data, we parsed hazard ratios for clinical efficacy and acceptability from Figure 4 in Cipriani et al. (2018). We manually mapped the 21 drugs to their DrugBank identifiers and organized the data into a .csv file which is available in our GitHub repository.

### Running PathFX on all drugs

Because we were running multiple analyses of drug effects, we generated a pipeline to analyze all drugs available in DrugBank version 5.1.6 using PathFX version 2 (released with Wilson et al., 2021). Version 2 contained data from 2020 and was an update of the original PathFX release in 2018. In brief, PathFX generates a PPI network around drug targets based on the amount and quality of evidence supporting the PPIs. To select network proteins, PathFX uses an empirical search of the entire scored network and selects an edgescore threshold value that maximizes the number of downstream proteins included in a target network that are above the average score in that target’s local neighborhood. This approach allowed for greater differentiation between target neighborhoods.

Next, PathFX uses a Fisher’s exact test to discover biological phenotypes associated with the drug’s network (full description in Wilson et al., 2018). For each drug network, PathFX assessed the overlap with a set of disease or side effect-associated proteins and used a Fisher’s exact test to assess the significance of the overlap. If a phenotype has significant overlap with a predicted drug network, it is only retained if the p-value is more significant than the median p-value of associations for 100 random drug networks generated with the same number of target proteins. After aggregating all significant associations, PathFX uses a Benjamini–Hochberg correction to control for multiple hypothesis testing and eventually filters out the least significant network phenotypes below the corrected p-value.

Importantly, using the empirical search and multiple filters for significant predictions allows PathFX to control for two biases in network methods. The first bias is related to high-degree, well-studied proteins for which PathFX contains a more stringent threshold for inclusion of these proteins (e.g., ubiquitin or p53) into a drug’s network. The second bias is related to phenotypes which have many annotated genes for which PathFX compares any predicted phenotype to the expected association for 100 random networks.

For drugs with targets that are connected to the interactome network, PathFX yielded a network of PPIs and an association table of enriched phenotypes. We provided all DrugBank IDs from version 5.1.6 as inputs to PathFX, which generated networks for 7,012 drugs, of which 4,599 are approved, with documented drug-binding proteins that were also connected to the PathFX interactome. In the GitHub repository, we provided instructions for cloning PathFX and example code for recreating this analysis and parsing the results to create the images and results presented here.

### Analysis of predicted psychiatric drugs

After running PathFX, we searched for any predictions related to five psychiatric conditions tracked in PathFX: “schizophrenia,” “paranoid schizophrenia,” “bipolar disorder,” “unipolar depression,” and “major depressive disorder.” Importantly, PathFX does not distinguish between clinically distinct diseases and instead catalogs “pathway phenotypes” curated from multiple sources (Wilson et al., 2018). Because PathFX is an exploratory tool, we retained all pathway phenotypes related to psychiatric diseases. PathFX contained additional pathway phenotypes related to psychiatry, but we focused our analysis to a select few.

Because PathFX reported predictions for any drug network as a tab-delimited file, we used pandas in Python to search for all drug-specific predictions and merged these predictions into one meta-table (Supplementary File S1). We also used pandas in Python to count the number of drugs per phenotype, the number of total drug–phenotype pairs (some drugs were associated with more than one phenotype), and the most common network genes used to predict a drug association. We provide code for this analysis in our GitHub repository.

### Analysis of drug ATC codes

Using the drugs predicted by PathFX, we extracted their ATC codes directly from DrugBank (Wishart et al., 2006; Law et al., 2014), retained all level-1 codes (the first character of each assigned ATC code—some drugs had multiple), and plotted these counts using Matplotlib in Python. ATC code information is provided by DrugBank and can be parsed from the “full_database.xml” object provided to registered users. To increase reproducibility, we provide example code for generating a .tsv file from the .xml file in our GitHub because we cannot release the raw DrugBank file to unregistered users.

### Visualization of PathFX networks

We used Cytoscape (version 3.10.0) (Shannon et al., 2003) to visualize the PathFX protein interaction networks and predicted phenotypes. Specifically, we assessed the network file for dihydromorphine (DB01565_merged_neighborhood__withDrugTargsAndPhens.txt) and the feature file (DB01565_network_nodeType.txt). Both files are default files produced by PathFX to enable network visualization. We have uploaded the Cytoscape session file to our GitHub to improve transparency.

### Running regularized regression using PathFX proteins and screen outputs

The PathFX analysis of all of DrugBank yielded a network file of PPIs for each drug. For all three screens, we obtained the relevant network files using their DrugBank identifiers and extracted the unique proteins from each file. We incorporated all network proteins into a binary matrix based on whether the protein (columns) was (1) or was not (0) in a particular drug network (rows).

We encoded the drug effect from each screen. For the computational screen, the effect was binary, with 1 representing an “increase” and 0 a “decrease” in phagocytosis. For the *in vivo* and clinical screens, the effect was continuous (i.e., the phenoscore or hazard ratio).

Then, using the drug-to-genes binary matrix as the input matrix and the effect column as the response column, we performed a ridge regression analysis (L2 regularization) using the “sklearn library” in Python. This was originally performed with a 70% training and 30% testing split of the drug rows and then later for exploratory analysis using the whole matrix or all drug rows. We provide code for each regularized regression analysis and code to regenerate the input matrices from the raw data in our GitHub repository.

We also implemented an analysis of coefficients using shuffled data. We used the same input data matrices and regression parameters, although we shuffled the effect columns (i.e., predicted effects on phagocytosis, phenoscore, or Efficacy O.R.S—we did not repeat this analysis for Acceptability O.R.S—using numpy.random.shuffle). For all three screens, we shuffled the data 100 times and reported the min, max, mean, and median coefficients generated with shuffled data. These are reported in Supplementary File S2.

After averaging coefficient scores for proteins identified in all three regression analyses, we also used pandas in Python to link drug screen scores with high regression scores. These summaries are now provided in Supplementary File S3.

### Plotting

We made all plots using Matplotlib in Python. We used a Seaborn clustermap for making heat maps of all network proteins and used venn2 to make Venn diagrams. We provide copies of all plotting code in our GitHub repository.

### Running GO enrichment

We used Enrichr (Chen et al., 2013), a Gene Ontology enrichment analysis tool developed by the Ma’ayan Laboratory using their web portal (https://maayanlab.cloud/Enrichr/). We assessed GO terms associated with network genes with relatively high or low regression coefficients. Because each screen had a unique number of genes and absolute coefficient values, we used slightly different thresholds for selecting input gene lists to GO enrichment. However, our goal was generally to identify the functions associated with top and bottom genes. In all cases, we used a background of all network genes in the PathFX network. For the *in silico* screen, which contained 1843 genes with regression coefficients, we selected the top 25% (any positive coefficients >0.20), and this yielded a list of 86 favorable genes. To keep gene lists of similar length, we selected the bottom 86 genes (any negative coefficients < −0.17). For the zebrafish screen, which has 2219 genes with regression coefficients, we simply selected the top 100 (any positive coefficients >0.013) and bottom (any negative coefficients < −0.011) 100 genes as favorable and unfavorable, respectively. For the antidepressant screen, which contained 324 genes with regression coefficients, we selected the top and bottom 5% (any positive coefficients >0.01 and any negative coefficients <0.01) as inputs to GO enrichment. This yielded 26 genes as “favorable” and 26 as “unfavorable.” For all comparative analyses, to account for multiple hypothesis testing, we used the “Adjusted p-value” as reported directly by Enrichr. According to their documentation, Enrichr uses the Benjamini–Hochberg (BH) method for calculating the “Adjusted p-value” reported in this study.

### Data availability

All code and data related to this project are included in https://github.com/jenwilson521/trans_psych_net. We included all results from PathFX analysis, regression analysis, and GO enrichment. We have not provided the raw data from Gravina et al. (2022), McCarroll et al. (2019), or Bruni et al. (2016), but we have provided instructions for accessing the data and code for parsing the raw data once obtained from the original source.

## Results

### A comparative pathway analysis to understand drugs with strong effects across screening platforms

**FIGURE 1:**
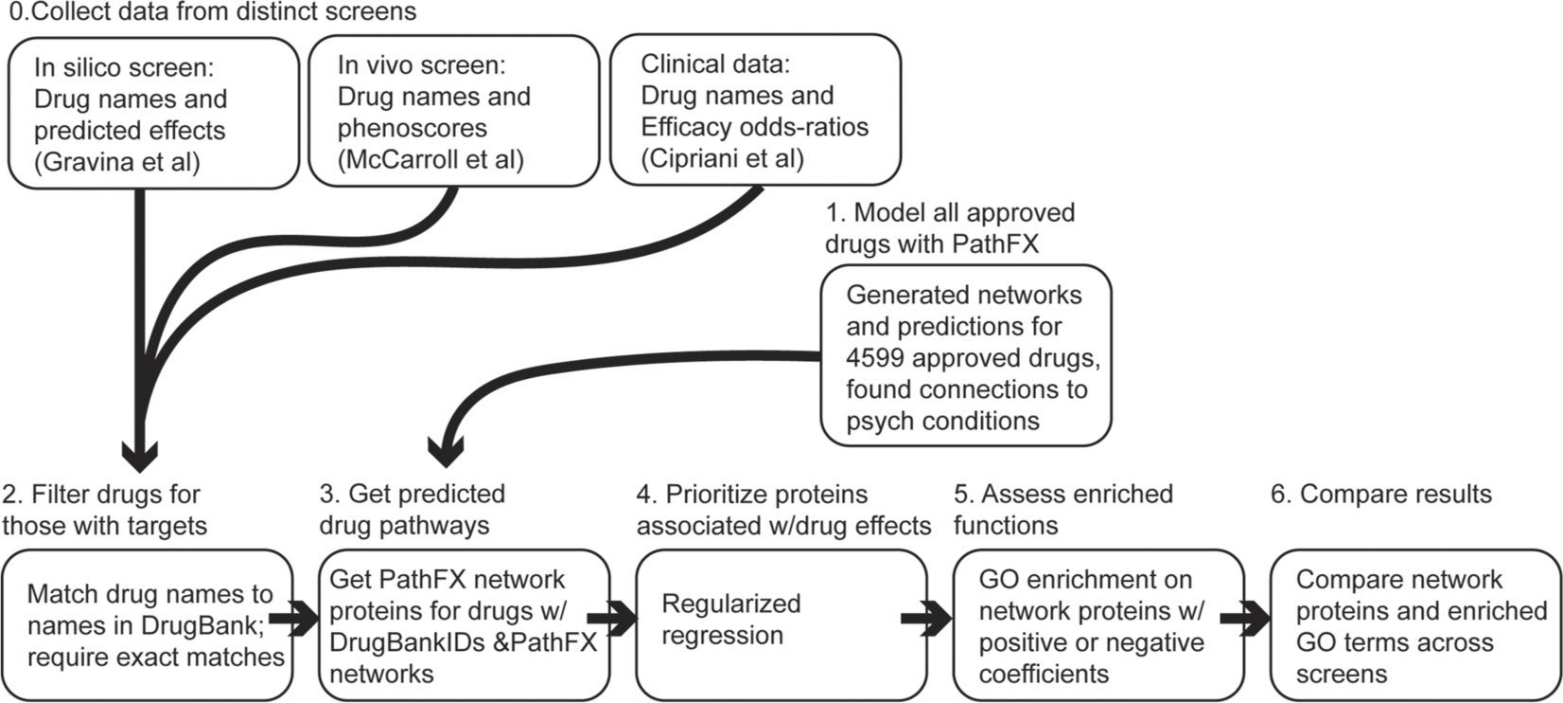
Parallel, pathway-based analysis of three distinct drug screens facilitates comparative assessment of possible shared mechanisms. Our workflow began with downloading drug and effect data from three screens. For all drugs, we required their names to have a direct match to a drug name in DrugBank so that we could consistently retrieve their drug targets and access their predicted pathways in PathFX. Separately, we modeled all approved drugs in PathFX and first investigated their predicted pathways and connections to psychiatric diseases. After filtering drugs and accessing their PathFX pathways, we used regularized regression on each dataset to understand which predicted pathways were associated with strong drug effects. We conducted Gene Ontology (GO) enrichment on proteins with high and low regression coefficients. Lastly, we compared network proteins prioritized by regression and GO terms across screens.

We hypothesized that pathway analysis of drugs tested across multiple screening platforms may find distinct features for drugs tested across unique screens. To test this, we collected data from three distinct “screens” in psychiatry: an *in silico* screen that predicted drug effects on microglia phagocytosis to advance candidates for schizophrenia (Gravina et al., 2022), an *in vivo* screen that tested drugs for paradoxical excitation in zebrafish (McCarroll et al., 2019), and a meta-analysis of the clinical efficacy and acceptability of antidepressants (Cipriani et al., 2018). We conducted parallel analysis of drugs from each of these studies by using their PathFX-predicted drug pathways and regression to identify which pathway proteins could explain drug effects and then compared whether pathways were similar across screes, despite being tested for different psychiatric applications. We also used GO functional enrichment to compare predicted drug effects. We outlined this approach in Figure 1.

### Pathway analysis uncovers broad associations with multiple psychiatric diseases

**FIGURE 2:**
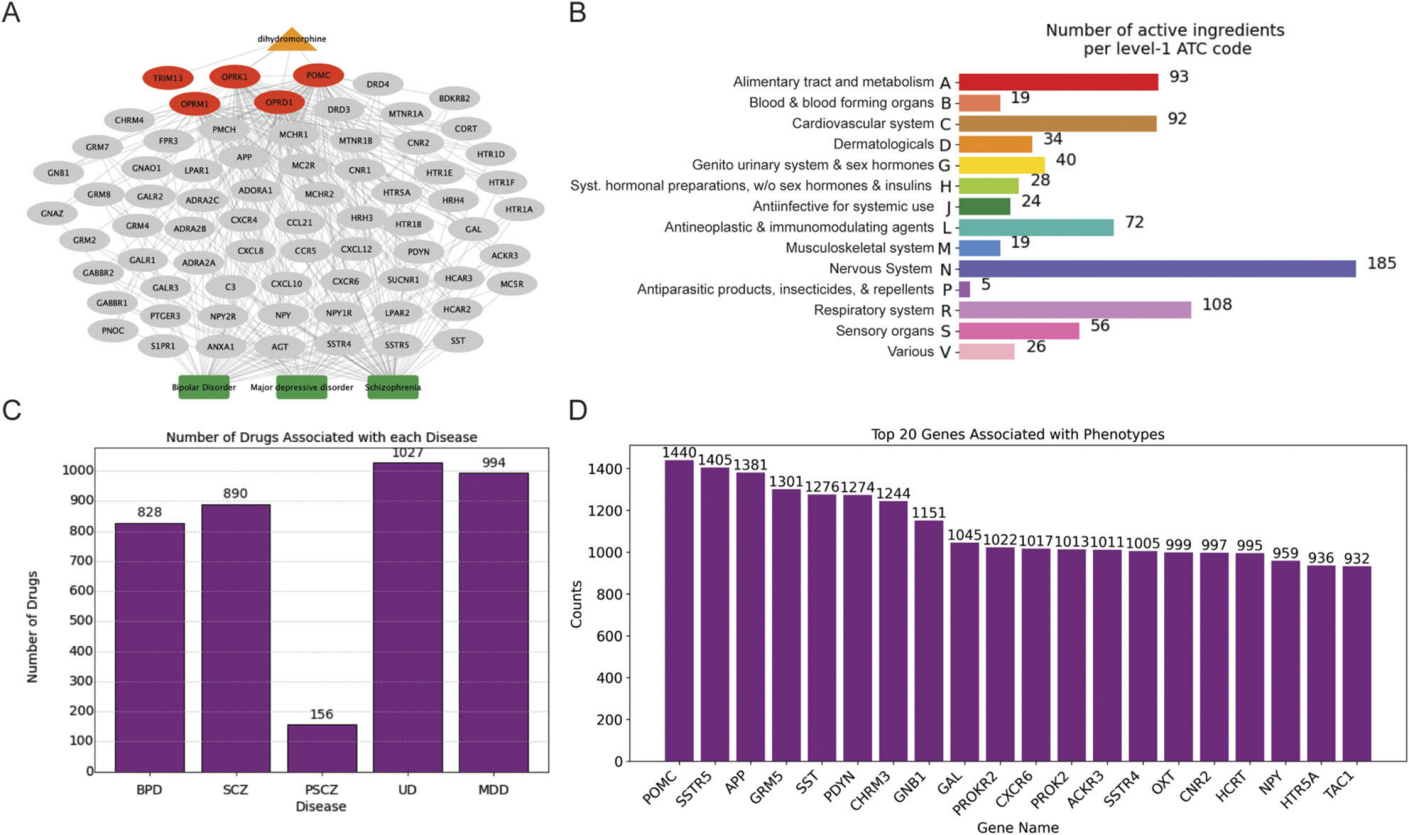
PathFX predicted approved drugs to have associations to multiple diseases. **(A)** PathFX connected dihydromorphine to multiple psychiatric and neurological disorders, with three highlighted. The drug, drug-binding target proteins, downstream proteins, and network-associated phenotypes are shown as an orange triangle, red ellipses, gray ellipses, and green boxes, respectively. Network edges are muted to highlight protein and phenotype names. **(B)** Count of level-1 ATC codes associated with 757 drugs predicted by PathFX. **(C)** Total unique drugs by disease area; BPD (bipolar disorder), SCZ (schizophrenia), PSCZ (paranoid schizophrenia), UD (unipolar depression), and MDD (major depressive disorder). **(D)** Network proteins ranked by the number of drug–phenotype relationships.

To generally understand drug compounds with pathways connected to psychiatry, we used the PathFX algorithm to predict associations between drugs in DrugBank and a set of five psychiatric diseases (unipolar depression, major depressive disorder, bipolar disorder, schizophrenia, and paranoid schizophrenia). In brief, PathFX discovers high-quality proteins downstream of drug targets for both approved and experimental drugs and network-associated pathway phenotypes (more details in Methods). PathFX selects high-quality proteins using a weighted interaction network where edges are weighted on the aggregate of published, experimental evidence supported the likelihood of an interaction while controlling for biases associated with highly studied proteins by using protein-specific evidence thresholds for inclusion in the final network. An example network is shown for dihydromorphine, an experimental, opioid derivative not currently used in patient treatment (Figure 2A), where PathFX connected the drug to 29 psychiatric phenotypes, of which three are shown (all other predictions in Supplementary File S1). PathFX predictions are driven by associations with the drug’s target proteins and other high-quality “downstream” proteins added by the algorithm (see Wilson et al., 2018 for more specific details). For instance, PathFX predicted a connection to “Schizophrenia” using 58 network proteins, including four drug targets—–the opioid receptors OPRK1, OPRM1, and OPRD1, and pro-opiomelanocortin (POMC)—and 54 additional downstream proteins, shown in gray. The downstream proteins are predicted to be part of the drug’s pathway. The drug’s target, TRIM13, which codes for the tripartite motif containing 13 proteins, is not associated with any predictions for psychiatric diseases.

In total, this analysis returned 2,245 total drug–phenotype relationships associated with five psychiatric conditions (all network predictions provided in Supplementary File S1). The drugs were predicted to have associations with psychiatric diseases and were assigned to several distinct therapeutic classes (Figure 2B). Unsurprisingly, most frequently PathFX predicted drugs intended for the neurological system (“N” code) (185 drug–disease pairs; some drugs may have more than one ATC code), and the algorithm predicted drugs intended for the “respiratory system” (“R” code, 108 drug–disease pairs), “alimentary tract and metabolism” (“A” code, 93 drug–disease pairs), and “cardiovascular system” (“C” code, 92 drug–disease pairs) as the next most frequent uses. These associations contained 757 unique drugs and 634 unique genes/proteins. Unipolar depression had the most predicted drugs (740), and schizophrenia had the fewest (416 drugs) (Figure 2C).

We next assessed the most frequently occurring network proteins in PathFX drug–phenotype predictions and observed that network predictions were supported by various pathway proteins (Figure 2D). Importantly, PathFX may use the same proteins to predict multiple drug–disease associations (e.g., pro-opiomelanocortin (POMC) was used to connect risperidone to “unipolar depression” and “major depressive disorder”). The most common protein-coding genes included G-protein subunit beta 1 (GNB1), POMC, somatostatin receptor subtype 5 (SSTR5), amyloid beta precursor protein (APP), oxytocin/neurophysin I prepropeptide (OXT), glutamate metabotropic receptor 5 (GRM5), hypocretin neuropeptide precursor (HCRT), neuropeptide Y (SPY), cholinergic receptor muscarinic 3 (CHRM3), and somatostatin (SST). Many of these pathway proteins have known connections to psychiatric disease, including serotonin receptors (Naughton et al., 2000), neuropeptide Y (Eaton et al., 2007), and oxytocin (Cochran et al., 2013). However, other connections, such as through the somatostatin receptors (SSTR5 and SSTR4), prokineticin and its receptor (PROK2 and PROKR2), and chemokine receptor 6 (CXCR6), were not obvious drivers of psychiatric diseases. This demonstrates PathFX’s ability to leverage established disease connections and exploratory associations to learn new drug effects. Overall, the pathways analysis demonstrated substantial pathway predictions and that, across predicted drugs, they shared several protein connections. In previous research using drugs screened for effects on microglia phagocytosis, we discovered that PathFX analysis of drugs with similar effects on microglia shared protein pathways (Bowen et al., 2023). We next wanted to understand whether PathFX could again identify shared pathways within and between multiple screens and clinical utility data. We specifically emphasized a computational screen, an *in vivo* screen, and published data about the effectiveness and acceptability of antidepressants in the clinic.

### A computational screen for phagocytosis inhibitors prioritized drugs with network associations to transporters, G-proteins, and GABA signaling

We first compared PathFX predicted pathways to drug effects from a published computational screen designed to identify repurposing candidates for schizophrenia (Gravina et al., 2022). To briefly summarize the approach from the published computational screen, the authors converted drug chemical strings to fingerprints and predicted their ability to inhibit or activate glial cell phagocytosis. The *in silico* screen was trained on experimentally tested drug compounds from Bowen et al. (2023), and then the algorithm was applied to predict the effects of untested chemical compounds. The original experimental screen was motivated by research connecting decreased synaptic density in the brain with the evolution of schizophrenia (Glausier and Lewis, 2013; Onwordi et al., 2020), suggesting that inhibitors of phagocytosis could be beneficial (“favorable”) treatments for multiple psychiatric diseases. Indeed, the original paper discovered that beta-2 adrenoceptor agonists induced protein-level changes in mouse models (Bowen et al., 2023), and the *in silico* screen discovered some support from the literature for its predictions. Interestingly, the chemical structure was insufficient to predict all drug effects. We hypothesized that multiple proteins could be involved in the regulation of phagocytosis and that pathway analysis could complement chemical structure–function-based predictions.

We explored data from Gravina et al. (2022) and selected drugs predicted to increase or decrease phagocytosis through their optimal machine learning approach (see their paper for more detail). Next, we used PathFX to model 285 drugs from the computational screen with documented drug-binding targets in DrugBank (Wishart et al., 2006; Law et al., 2014). PathFX requires drug target information, and not all the screened molecules had documented target proteins in DrugBank. Of these drugs, 81 and 204 were predicted to increase or decrease phagocytosis, respectively.

**FIGURE 3:**
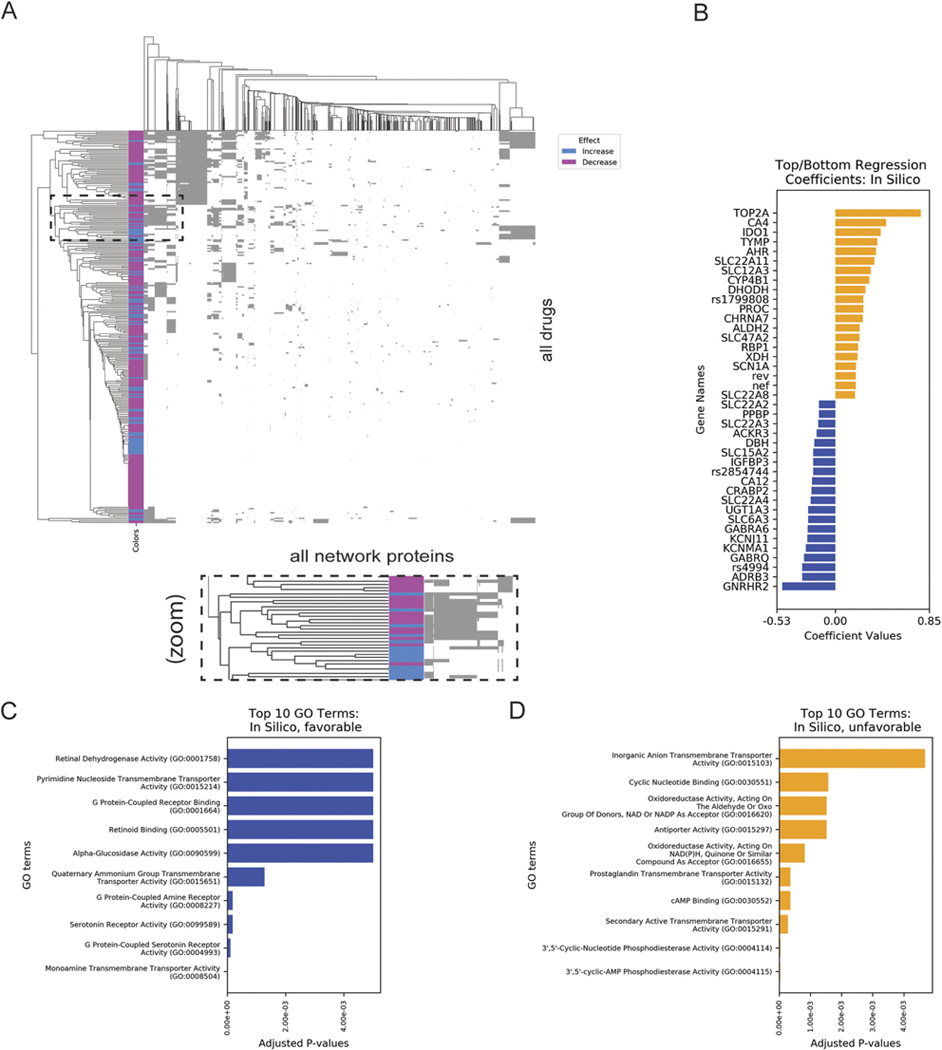
PathFX predictions discovered shared pathways among drugs with unique predicted effects on phagocytosis. **(A)** Drugs (rows) are plotted against network proteins (columns). Gray shading indicates the presence of a protein in a drug’s network. Pink/blue row indicate whether the drug was predicted to increase/decrease phagocytosis. Inset highlights one group of drugs that share predicted network proteins despite having distinct effects on phagocytosis. **(B)** Regression coefficients for network proteins associated with increased or decreased phagocytosis are plotted in orange or blue, respectively. Top enriched GO terms for proteins with low **(C)** or high **(D)** regression coefficients.

To better understand network patterns among drugs with similar effects, we used logistic regression to prioritize drug network proteins associated with decreased (“favorable”: expected to reduce synaptic pruning and disease symptoms) or increased (“unfavorable”: expected to increase synaptic pruning and aggravate symptoms) phagocytosis. We plotted the binary matrix, which is color-coded by drug effect (Figure 3A, matrix in Supplementary Figure S2). The sparsity of the matrix demonstrates the general dissimilarity between compounds and their predicted pathways. Notably, drugs with dissimilar effects on phagocytosis (shown as pink or blue on each row) clustered together, suggesting that their effects on phagocytosis could be mediated by altered activating or inhibitory effects on the same network proteins.

We used regularized logistic regression to determine whether PathFX-predicted pathway proteins were associated with the drug’s simulated effect on phagocytosis. We then analyzed regression coefficients to understand patterns (top genes in Table 1; Figure 3B, all other genes in Supplementary Figure S2). Separately, the logistic regression was not predictive in a train/test split (mean-squared error = 0.12, coefficient of determination = 0.4), suggesting that network proteins were also insufficient to explain all drug effects.

Our analysis discovered multiple pathway proteins associated with increased and decreased phagocytosis, some of which have associations with psychiatric disease in the literature. Among the top “unfavorable” genes (associated with predicted increases in phagocytosis), we discovered DNA regulators like DNA topoisomerase II alpha (TOP2A, coeff = 0.77), pharmacokinetic proteins such as solute carrier family 22 member 11 and family 12 member A3 (SLC22A11, coeff = 0.35, SLC12A3, coeff = 0.32), the zinc metalloenzyme, and carbonic anhydrase 4 (CA4, coeff = 0.46). Previous studies have discovered an association between TOP2B (a family member of TOP2A) and neurodevelopmental disorders associated with schizophrenia (Harkin et al., 2016). A case report was found in which a patient diagnosed with schizophrenia-like psychosis had a homozygous mutation of exon 23 in the SLC12A3 gene (Pan et al., 2016).

Among the genes with low or “favorable” regression coefficients, we identified the potassium calcium-activated channel subfamily M alpha 1 (KCNMA1, coeff = −0.27, Table 1). Previous studies found that KCNMA1 has a common gene signature with a link to epilepsy, movement disorders, and wide paroxysmal neurological presentations (Ilyas et al., 2020). Additionally, a meta-analysis discovered that some polymorphisms in solute carrier family 6, member 3 (SLC6A3, coeff = −0.25), which codes for the dopamine transporter, might be risk factors for schizophrenia or linked to schizophrenia (Xu et al., 2020). Finally, additional studies discovered that downregulated insulin growth factor binding protein 3 (IGFBP3, coeff = −0.42) was associated with schizophrenia and other phenotypes such as intellectual disability and hypotonia (Perez et al., 2018).

To better understand drug-associated pathways, we performed GO enrichment on the network genes with the most extreme coefficients. To keep the analysis to a manageable size, we took the top 86 (coeff >0.20) and bottom 81 (coeff < −0.17) genes from the logistic regression mentioned above (Supplementary Figure S2). We used Enrichr (Chen et al., 2013) and retained the top ten GO terms for network proteins associated with decreased (Figure 3C) and increased (Figure 3D) phagocytosis. This analysis yielded enrichment to 143 and 153 terms for the favorable and unfavorable network proteins, respectively. Among all terms, regardless of significance, we observed associations with g-protein signaling and neurotransmitter signaling (Figures 3C,D, select terms in Table 2, all terms in Supplementary Figure S2).

We also discovered support from the literature for many of the biological processes found through the GO enrichment of network proteins associated with unfavorable effects on phagocytosis. For instance, the term “G-protein-coupled serotonin receptor binding” was supported by multiple studies that described a relationship between serotonin receptor antagonism and treatment for schizophrenia (Reynolds, 2004; Miyamoto et al., 2012; Ceskova, 2022). Furthermore, the term “cyclic AMP binding” is related to the fact that there is an increased amount of cyclic AMP in schizophrenia patients, following the dopamine theory that schizophrenia is linked to increased dopaminergic function and, therefore, more cyclic AMP. Another term associated with the unfavorable network proteins was “amyloid-beta binding,” which is well documented for its role for Alzheimer’s disease (Hampel et al., 2021) but is supported by Dean et al. (2024) who found lower levels of soluble precursor, but not the full protein, in patients with schizophrenia.

### An *in vivo* screen prioritized drugs associated with transporter and metabolizing enzymes

Ideally, animal models of psychiatric disease complement *in silico* approaches for identifying novel drug treatments, and we hypothesized that strong candidates from either approach would share predicted pathway effects. To make this comparison, we analyzed an *in vivo* dataset from two related publications: Bruni et al. (2016) developed a battery of *in vivo* behavioral assays in zebrafish screens for the purposes of identifying novel treatments for psychiatric diseases such as schizophrenia, and McCarroll et al. (2019) leveraged established behavioral assays to study paradoxical excitation—the ability of sedative drugs to stimulate activity in zebrafish. Using haloperidol-induced behavioral profiles, the authors identified additional compounds with potential applications in psychiatric treatment by quantifying behavior across assays and reporting a summary phenoscore. In this framework, higher coefficients were associated with higher phenoscores and were indicative of drugs with a higher potential of repurposing.

**FIGURE 4:**
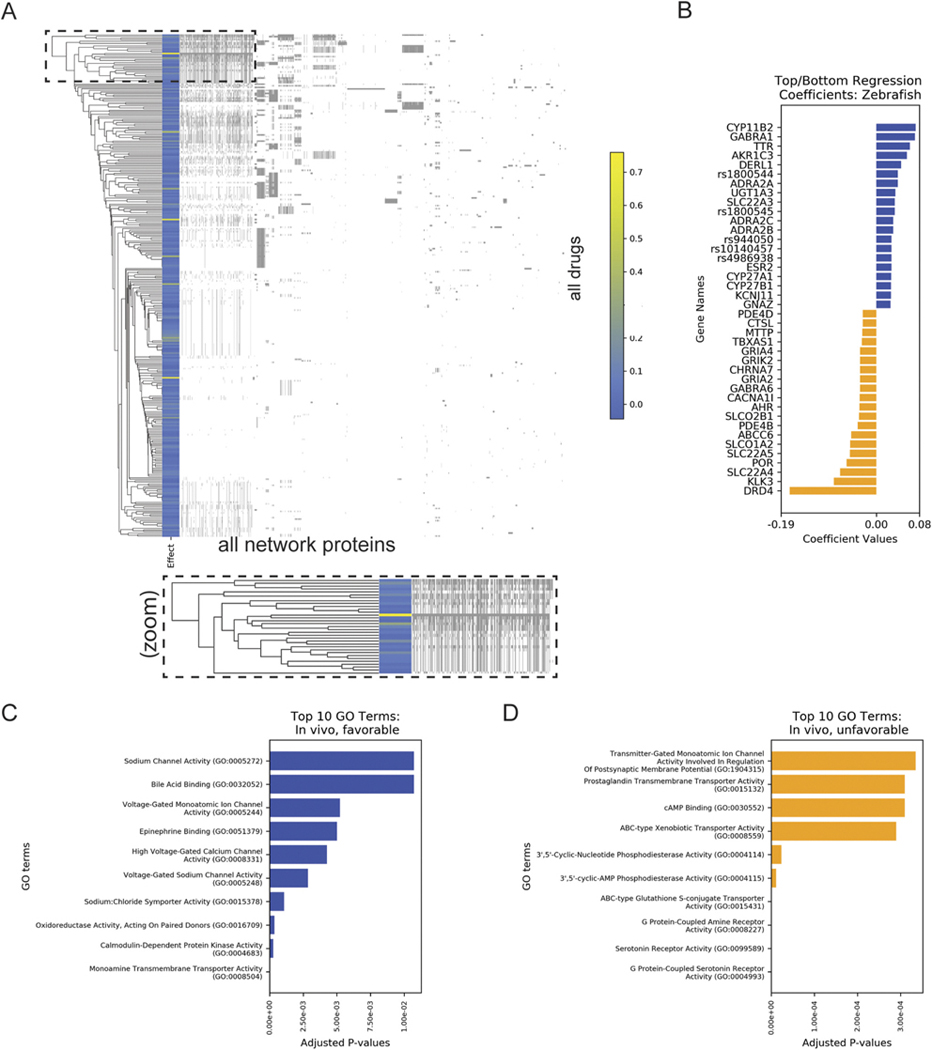
PathFX predictions discovered shared pathways among drugs with a range of excitatory effects in zebrafish. **(A)** Drugs (rows) are plotted against network proteins (columns). Gray shading indicates the presence of a protein in a drug’s network. Blue → yellow rows indicate the drug’s phenoscores, with yellow or blue indicating a higher or lower score, respectively. The inset highlights that drugs with relatively high phenoscores (yellow rows) share similar network proteins with drugs with lower phenoscores (blue rows) **(B)** The regression coefficients for network proteins associated with high or low phenoscores (reported in (22)) are plotted in blue or orange, respectively. Top enriched GO terms for proteins associated with favorable **(C)** or unfavorable **(D)** drug networks. The y-label for GO term GO:0016709 in **(C)** was shortened from “oxidoreductase activity, acting on paired donors, with incorporation or reduction of molecular oxygen, NAD(P)H as one donor, and incorporation of one atom of oxygen (GO:0016709)” to better fit into the figure frame.

We again used PathFX analysis to model 327 drugs from the screening data (all drugs listed in Supplementary Figure S2 and network proteins highlighted in Figure 4A) and further used regularized linear regression to understand the association of the PathFX pathway proteins with the phenoscore measured in McCarroll et al. (2019) (top coefficients in Table 3; all other genes in Supplementary File S2). Like the *in silico* screen, the heatmap of all network proteins was sparse, and the relatively few high-scoring drugs (rows highlighted in yellow) were distributed throughout the heatmap (Figure 4A). This highlights that a single pathway could not describe all drugs with high phenoscores. Compared to the *in silico* screen, the coefficients were overall lower, suggesting that drug networks with similar effects on zebrafish excitation used distinct pathways. The individual genes in the top/bottom ten were also distinct from those in the *in silico* screen (Figure 4B), although a few protein families were similar. Specifically, the SLC transporters were recovered in the top and bottom coefficients from the zebrafish screen, suggesting that drugs with distinct pharmacokinetic features could distinguish effects on zebrafish. The gamma-aminobutyric acid receptors (GABARs) and adrenoceptor families were recovered in both screens, with the GABA receptors GABRQ and GABRA6 being unfavorable in the *in silico* screen and the GABA receptor GABRA1 being associated with favorable drug outcomes in the zebrafish screen. In their original publication, McCarroll et al. (2019) note the relevance of GABAR targeting for paradoxical excitation, suggesting that PathFX-identified pathways were relevant to this system. The adrenoceptors (ADRs) were also implicated in both, with ADRB3 being favorable in the *in silico* screen and ADRA2A, ADRA2B, and ADRA2C being favorable in the zebrafish screen.

Again, we found evidence in the literature for the role of these network proteins in psychiatry. ADRA2A regulates cortical interneuron migration, which is connected to the adrenergic system (Riccio et al., 2012), and this receptor is present in central and peripheral nervous system tissues in zebrafish (Ruuskanen et al., 2005). Independently, our prior work had also discovered and tested the relevance of beta-2 adrenoceptor agonists for their utility as repurposing candidates for schizophrenia (Bowen et al., 2023). Because PathFX does not include directionality in its predictions, it is possible that the adrenoceptors are associated with unfavorable outcomes if the drug antagonized the receptors.

As before, we also assessed the GO functional enrichment of the top and bottom regression coefficients. Generally, the GO terms associated with the favorable drug networks were dominated by pharmacokinetic effects and highlighted the role of transmembrane proteins and transporters and of ion channels (selected terms in Figures 4C,D; Table 4, all other terms in Supplementary Figure S2). The unfavorable network proteins were associated with terms related to a shorter and distinct set of GO terms, including those related to G-protein activity, cAMP binding, ion channel activity, and phosphodiesterase activity (selected terms in Table 4, all other terms in SF2). The importance of pharmacokinetics is well-established in psychiatry; these are well reviewed in one study about the optimal blood dosing ranges for at least four antipsychotics (Mauri et al., 2018). The relevance of serotonin receptor g-protein activity is interesting because agonists of these receptors have antidepressant-like effects in mice (Pogorelov et al., 2023), suggesting that the zebrafish assay may be detecting drugs that inhibit this signaling cascade. The importance of phosphodiesterase 4 is well-established in the field (Zoubovsky et al., 2014), with other research supporting its psychiatric importance in the context of the gene, discovered in schizophrenia (DISC1) (Brandon and Sawa, 2011; Porteous et al., 2011), though the exact therapeutic role of this protein seems inconclusive. Overall, this suggests that pathways analysis leveraged disease-relevant proteins and that there were similarities, if not direct overlaps, among pathways associated with drugs from the *in silico* and *in vivo* screens.

### Pathway analysis of antipsychotic drugs used in the clinic prioritized drug pharmacokinetic and G-protein signaling effects

**FIGURE 5:**
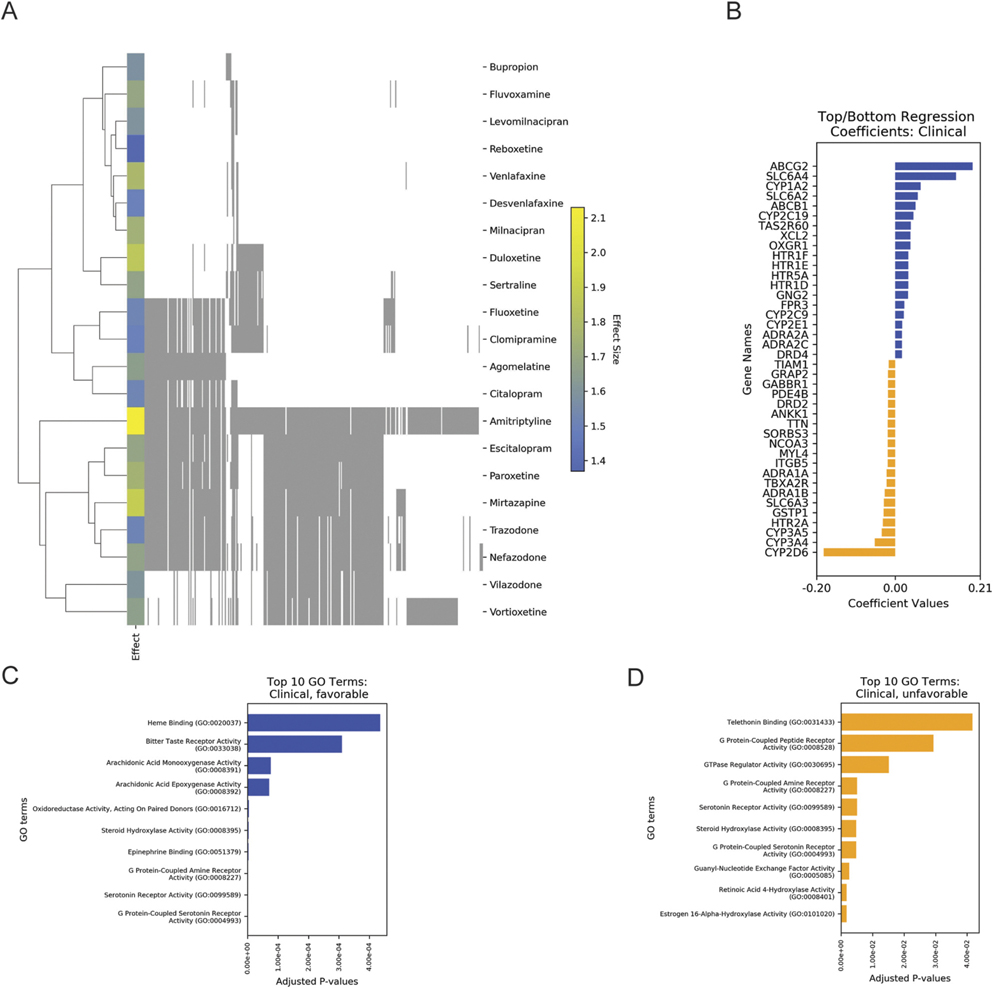
PathFX predictions discovered shared pathways among drugs with distinct efficacy and acceptability. **(A)** Drugs (rows) are plotted against network proteins (columns). Gray shading indicates the presence of a protein in a drug’s network. Blue → yellow row indicates the increasing efficacy (odds ratio) of the drug (Cipriani et al., 2018) **(B)** Regression coefficients for network proteins associated with higher or lower efficacy scores are plotted in blue or orange, respectively. Top enriched GO terms for proteins associated with favorable **(C)** or unfavorable **(D)** drug networks. Y-label for GO:0016712 in **(C)** was shortened from “Oxidoreductase activity, acting on paired donors, with incorporation or reduction of molecular oxygen, reduced flavin or flavoprotein as one donor, and incorporation of one atom of oxygen (GO:0016712)” to better fit into the figure frame.

We lastly compared PathFX-predicted pathways to antidepressant effects in the clinic. Specifically, we leveraged a meta-analysis that quantified the efficacy and acceptability of 21 antidepressants studied across 522 clinical trials (Cipriani et al., 2018). The meta-analysis reported overall efficacy and acceptability scores, quantified by an odds ratio, for all compounds (all compounds listed in Supplementary File S2). We again applied PathFX and regression analysis to identify relevant pathway proteins associated with antidepressants clinical scores. Like before, we used a heatmap to examine network proteins (Figure 5A) and identified more similarity in network proteins among clinically used drugs than in drugs used in exploratory screens.

We applied a regularized linear regression model to identify relationships between PathFX-identified drug network genes and clinical outcomes such as efficacy and acceptability. After performing these two methods, we found similar model coefficients regardless of using efficacy or acceptability data. As in the zebrafish screen, the coefficients were lower than those observed in the *in silico* screen, and the highest-weighted proteins were largely associated with drug pharmacokinetic effects (Figure 5B, top coefficients in Table 5, all other genes in Supplementary File S2). Specifically, the ATP-binding cassette family genes, serotonin-binding genes, and cytochrome enzyme genes (e.g., *ABCG2*, *SLC6A4*, and *CYP1A2*) had the highest and cytochrome P450 family member genes (e.g., *CYP2D6*, *CYP3A4*, and *CYP3A5*) had the lowest coefficients in the model. In applying regularized linear regression to clinical acceptability data, cytochrome P450 family enzyme genes, serotonin-binding genes, and isozyme genes (e.g., *CYP2D6*, *HTR2A*, and *GSTP1*) had the highest, and ATP-binding cassette family genes, potassium channel genes, and forming ligand-gated ion channel genes (e.g., ABCG2, KCNH2, and CHRNB4) had the lowest model coefficients. Only a few favorable network proteins, including the oxoglutarate receptor 1 (OXGR1), X-C motif chemokine ligand 2 (XCL2), and 5-hydroxytryptamine receptor 1D (HTR1D), and unfavorable network proteins, 5-hydroxytryptamine receptor 2A (HTR2A) and glutathione S-transferase pi 1 (GSTP1), adrenoceptor alpha 1B and 1A (ADRA1B and ADRA1A), and integrin beta-5 (ITGB5), suggest any differential pharmacodynamic effects. The adverse effects of adrenoceptor alpha 1 blockage was established many years earlier (Feighner, 1999); however, the same blockage is attributed to the antipsychotic effects of some compounds, including clozapine (Svensson, 2003). There is limited evidence for OXGR1, with one study citing ultra-rare mutations in the related gene as being associated with alcohol-use disorder (Hill and Hostyk, 2023). Similarly, there is limited information for the role of ITGB5, though one study discovered significant overexpression in schizophrenia and bipolar disorder patients compared to their non-effect relatives (Chagnon et al., 2020).

Again, we assessed GO terms associated with favorable and unfavorable network proteins (selected terms in Figures 5C,D; Table 6, all other terms in Supplementary Figure S2). We again observed many processes associated with drug pharmacokinetics and related to G-protein activity. Additionally, we saw terms related to serotonin receptors associated with both favorable and unfavorable network proteins, and terms related to G-protein binding and opioid receptor binding being specifically associated with the favorable networks. Furthermore, neurotrophin and neuropeptide binding were both associated with the networks of favorable drugs and the G-protein peptide receptor was associated with the networks of unfavorable, or relatively less clinically effective, drugs.

### Distinct psychiatric screening platforms select for drugs with similar predicted pathways

**FIGURE 6:**
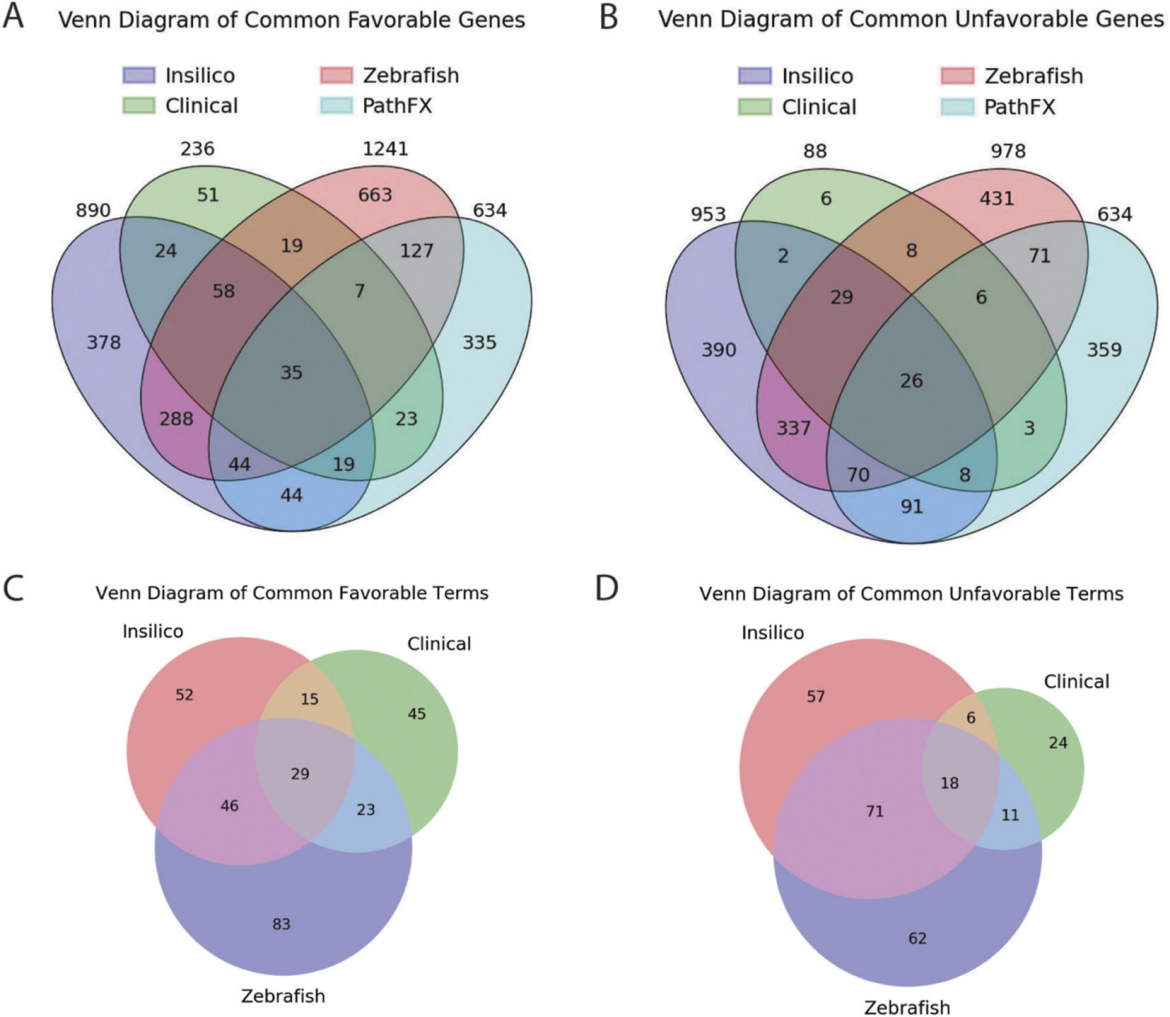
Network proteins and GO functions are shared across screening platforms. We computed the number of shared favorable **(A)** and unfavorable **(B)** network proteins (all PathFX proteins were included in both because they were not separated by “favorable” or “unfavorable”) and shared favorable **(C)** and unfavorable **(D)** GO terms assessed for the *in silico* and zebrafish screens and clinical data.

We first compared network genes that were considered “favorable” or “unfavorable” across all three screens with any PathFX-identified gene/protein (see Methods); we discovered 35 and 26 genes prioritized as favorable or unfavorable, respectively, that also overlapped with at least one PathFX prediction, and 92 and 55 genes prioritized as favorable or unfavorable, respectively, if considering overlap among screens only (Figures 6A,B; Supplementary Figure S2). Overall, the shared network proteins had relatively low regression coefficients across each screen, yet this was not surprising given that the absolute differences between drug effects were relatively small. Despite small coefficient values across all screens, these were still an order of magnitude larger than coefficients generated with shuffled data (Supplementary Figure S2). Among the network genes with the strongest favorable associations, the transporters, serotonin receptors, adrenoceptors, and adenosine receptors had the highest average scores across the screens (Table 7). Generally, the top unfavorable shared genes included two serotonin and one GABA and glutamate receptor, an adrenoceptor, a g-protein and a gastrin-peptide associated gene (Table 7). Otherwise, the remainder of the network proteins had relatively small average coefficients, suggesting that they had minor predictive utility for discerning drugs with unfavorable effects.

Regardless of average rank, we found support in the literature for the relevance of these proteins. For instance, multiple studies found that vasoactive intestinal peptide (VIP) mutations are associated with schizophrenia (Chen et al., 2022) and that the receptor for this molecule might be a viable drug target for the disease (Ago et al., 2021). The literature also supports the chemokine ligands in psychiatric disease, specifically that serum concentration of these ligands and their receptors are associated with posttraumatic stress disorder (Ogłodek et al., 2015). There is also interesting support in the literature for the upregulation of alpha-2 adrenoceptors in schizophrenic patients after antipsychotic drug treatment (Brocos-Mosquera et al., 2021).

Although our platform cannot discern directionality, we conducted an exploratory analysis of how the shared network proteins were associated with “top” drugs from each screen (Supplementary Figure S3). Broadly, we observed that top-scoring drugs using clinical efficacy or phenoscores shared similar drug targets and network proteins, even though most of the drugs were distinct. For instance, sertraline had a relatively high clinically-reported efficacy odds ratio (1.67) and a relatively high phenoscore (0.211). Regression of the clinical and zebrafish data both prioritize sertraline’s network proteins, SLC6A4 and DERL1, albeit with different absolute coefficient values. Sertraline is a known inhibitor of the sodium-dependent serotonin transporter SLC6A4, as documented in DrugBank (Wishart et al., 2006; Wishart et al., 2017), the Therapeutic Target Database (TTD) (Zhou et al., 2023), and other literature (Benmansour et al., 2002). On the other hand, DERL1 is a PathFX-predicted protein in sertraline’s network. DERL1 participates in a 37-protein complex with albumin, with which sertraline directly binds. DERL1 is part of a family of endoplasmic reticulum-associated degradation proteins, the dysfunction of which is associated with impaired neurogenesis and cognitive dysfunction in mice (Murao et al., 2024). To our knowledge, no connection has been discovered between sertraline and DERL1; however, our brief literature search suggests that sertraline’s efficacy may be associated with stabilized or enhanced DERL1 function, since loss of DERL1 is associated with cognitive dysfunction. This suggests that future efforts to infer directionality in our networks may uncover activating and inhibitory relationships within the same drug networks.

Taken together, this suggests some consensus across screens, despite distinct screening platforms and distinct outcomes. Furthermore, it suggests that methods converge on pathways with known associations with psychiatric disease, even if the exact disease areas are not consistent.

As with the genes, we also assessed shared favorable and unfavorable GO terms across screening platforms. Overall, there were three favorable (“monoamine transmembrane transporter activity,” “sodium:chloride symporter activity,” and “steroid hydroxylase activity”) GO terms and one unfavorable (“serotonin receptor activity”) GO term that significantly associated with each gene set across all three screens (Table 8). Interestingly, the shared biological functions were associated with distinct network proteins, suggesting that predicted drug pathways may “smooth out” differences in screening platforms. There were two favorable (“G-protein-coupled receptor binding” and “epinephrine binding”) and six unfavorable (“3′,5′-cyclic-AMP phosphodiesterase activity,” “3′,5′-cyclic-nucleotide phosphodiesterase activity,” “cAMP binding,” “cyclic nucleotide binding,” “G-protein-coupled serotonin receptor activity,” and “G-protein-coupled amine receptor activity”) GO terms that were statistically significant in at least two screens (Table 8). In total, 29 favorable and 18 unfavorable GO terms were shared, regardless of significance level (Supplementary File S2).

Again, we sought validation in the literature of these predictions and found sources to support their relevance to disease. Steroid hydroxylase is a class of hydroxylase enzymes involved in the biosynthesis of steroids. One study found that the CYP21A2 gene, which encodes for steroid 21-hydroxylase, is associated with risk for late-life depression—specifically, depression in women (Ancelin et al., 2021). Many studies, reviewed in Ren et al. (2014), support a connection between abnormalities of cyclic AMP response element binding function and pathophysiology in bipolar and schizophrenia illnesses.

## Discussion

Psychiatric diseases, including depression and schizophrenia, are chronic conditions with insufficient therapeutic options for patients. Drug discovery leverages multiple screening platforms to advance new candidates, including computational screens, omics data analysis, *in vivo* model systems, and historical analysis of clinical effects. However, with increasing levels of drug attrition and increased costs associated with development, the extent to which these platforms complement each other or how they may be better utilized to advance novel candidates is unclear. Given increasing evidence that modeling may overcome interspecies differences, we hypothesized that pathways modeling could overcome the challenge of bridging results across psychiatric model systems. Here, we pursued parallel pathway analyses as a means of finding themes among top candidates prioritized from a computational screen, *in vivo* screen, and clinical efficacy data, with an emphasis on schizophrenia and depression. Despite leveraging different experimental or clinical data, the pathways for associated “favorable” or “unfavorable” drugs not only shared pathway connections but also core molecular functions. Taken together, this suggests that despite unique connections (e.g., drug targets or pathway proteins), the meta-analysis of pathway effects predicts similar cellular changes as favorable or unfavorable.

After analysis of three screens, their shared gene pathways, and their shared Gene Ontology terms, we observed that the three screens were more similar than we first anticipated. We found the level of overlap promising, especially because the screens were designed without shared goals. Indeed, an *in silico* screen for structure–function relationships in astrocytes is biologically distinct from an excitation screen in zebrafish, and moreover, both systems are distinct from human patients. Looking at enriched GO terms and their associated network proteins, our analysis suggests that favorable drugs in psychiatry may influence g-proteins, solute carrier family proteins, adrenoceptors, and steroid hydroxylase activity. Our results also suggest that unfavorable drugs affect serotonin receptors and phosphodiesterase activity.

Despite studying clinically distinct diseases, the literature supports the insight that psychiatric disease may share similar changes in cellular function. In our pathways analysis, we found substantial evidence of drug connections with psychiatric diseases, and our psychiatric diseases often co-occurred within the same drug networks. In the PathFX database, which aggregates associations from ClinVar (Landrum et al., 2014), Online Mendelian Inheritance in Man (OMIM) (Hamosh et al., 2002), Phenotype-Genotype Integrator (Ramos et al., 2014), and DisGeNET (Piñero et al., 2015; Piñero et al., 2017), many psychiatric diseases share molecular characteristics (i.e., disease-associated genes), and the literature supports these shared features. As an example, genetic analysis of people of European ancestry revealed that multiple variants in AKT1 increase the risk for both schizophrenia and bipolar disorder (Karege et al., 2012). Bipolar disorder (BD) and schizophrenia (SZ) share genetic susceptibility, and polygenic risk scores distinguished patients with both BD and SZ (Calafato et al., 2018). Relatedly, when using polygenic risk scores to discern BD, SZ, MDD, and Parkinson’s Disease (PD), even larger polygenic risk scores were insufficient for discerning differences between the diseases (Schulze et al., 2014), further emphasizing their shared molecular etiology. Moreover, deficiencies in regulatory biology, specifically mRNA regulation and protein translation, are altered across psychiatric diseases (Laguesse and Ron, 2020).

There are several limitations to this study. Importantly, in the PathFX approach, the networks do not include directionality. An association with an unfavorable gene does not indicate whether the drug activates or deactivates the gene, or if this connection has a favorable or unfavorable outcome for the predicted disease. Without pathway “activity,” we may miss shared or unique effects of drugs from each screen type. This presents additional challenges for pharmacology because we cannot establish preferences for agonists or antagonists or whether pathway biomarkers may be associated with preferred clinical outcomes. This limitation is highlighted by our exploratory analysis of sertraline, where we hypothesize that its “efficacy” is due to inhibition of its serotonin transporter, SLC6A4, but activation or stabilization of its downstream protein, DERL1. This indicates that future efforts to understand pathway “activity” will need to comprehensively study activating and inhibitory relationships within a drug’s pathway. Additionally, PathFX networks and predictions are only partially validated, due to insufficient data for this effort. We and others have discussed insufficient examples of drug true negative effects as a limitation for advancing network methods generally (Wilson et al., 2022a; Harpaz et al., 2014). However, we are currently devising new approaches to test PathFX predictions, including our previous application in schizophrenia drug discovery (Bowen et al., 2023), another two studies in drug-induced side effects (Alidoost and Wilson, 2024; Wilson et al., 2022b), and one more in neurodegeneration (Alidoost et al., 2024). Interestingly, we have discovered that comparisons of PathFX “over-predictions” with phenotypic and clinical data have rendered many predictions as true positives.

Lastly, another limitation that also presents an opportunity is that we only considered three screens in this study. However, we discovered similar gene functions associated with drugs with strong effects. This suggests an opportunity to expand the analysis more comprehensively to a greater number of drug screens. Additionally, as deep learning approaches become more widespread in drug development, we anticipate a need for careful selection of drug features to build performant algorithms with higher translational potential. Our analysis suggests that gene functional information of predicted drug pathways may be of high utility to building these algorithms. Future efforts could again assess shared GO functional terms associated with drug screens in psychiatry and later use these features to build more performant and predictive algorithms.

The direct integration of pathways modeling may improve therapeutic development efforts in general, especially given high drug attrition and deficiencies noted in many model systems. Interestingly, a review of target-discovery pipelines (Plenge et al., 2013) recounted the challenges with relating human biology to animal models or developing model systems to understand mechanisms when human data (e.g., epidemiological studies) supported a development program. We and others have discovered that computational models may “smooth out” inconsistencies across translational platforms. As mentioned previously, Brubaker et al. (2019) discovered that signaling components could find consensus understanding between mouse and human samples. Related to this, network models of cell- and tissue-specific model organisms have successfully recovered disease genes for human conditions (Yao et al., 2018), again demonstrating that a pathways perspective can bridge animal-to-human translational efforts. Taken together, this underscores the value of pathways meta-analysis for facilitating translation across distinct developmental platforms and building performant algorithms that advance drug candidates with high translational potential.

## Supplementary Material

SF3

SF2

SF1

The Supplementary Material for this article can be found online at: https://www.frontiersin.org/articles/10.3389/fddsv.2025.1686789/full#supplementary-material

## Figures and Tables

**TABLE 1:** PathFX network proteins with low (“favorable,” decreased phagocytosis) or high (“unfavorable,” increased phagocytosis) coefficients from logistic regression.

Bottom 10 genes and regression coefficients (“favorable”)		
Gene symbol	Gene name	Regression coefficient
*GNRHR2*	Putative gonadotropin-releasing hormone II receptor	−0.48
*ADRB3*	Adrenoceptor beta 3	−0.30
*rs4994*	β3-adrenoceptor polymorphism	−0.30
*GABRQ*	Gamma-aminobutyric acid type-a receptor subunit theta	−0.28
*KCNMA1*	Potassium calcium-activated channel subfamily M, alpha-1	−0.27
*KCNJ11*	Potassium inwardly rectifying channel subfamily J, member 11	−0.25
*GABRA6*	Gamma-aminobutyric acid receptor subunit alpha-6	−0.25
*SLC6A3*	Solute carrier family 6, member 3	−0.24
*UGT1A3*	UDP-glucuronosyltransferase 1A3	−0.24
*SLC22A4*	Solute carrier family 22, member 4	−0.22
Top 10 genes and regression coefficients ("unfavorable”)		
Gene symbol	Gene name	Regression coefficient
*TOP2A*	DNA topoisomerase II alpha	0.77
*CA4*	Carbonic anhydrase 4	0.46
*IDO1*	Indoleamine 2,3-dioxygenase 1	0.41
*TYMP*	Thymidine phosphorylase	0.38
*AHR*	Aryl hydrocarbon receptor	0.37
*SLC22A11*	Solute carrier family 22, member 11	0.35
*SLC12A3*	Solute carrier family 12, member 3	0.32
*CYP4B1*	Cytochrome P450 4B1	0.31
*DHODH*	Dihydroorotate dehydrogenase	0.27
*PROC*	Protein C, inactivator of coagulation factors Va and VIIIa	0.25

**TABLE 2:** Select example GO terms emphasizing G-protein and cell signaling processes.

Term	Adjusted P-value	Gene
Favorable		
G-protein-coupled serotonin receptor activity (GO:0004993)	1.11E-04	*HTR1E*; *HTR1F*; *HTR1D*; *HTR5A*
G-protein-coupled amine receptor activity (GO:0008227)	1.86E-04	*HTR1E*; *HTR1F*; *HTR1D*; *HTR5A*
G-protein-coupled receptor binding (GO:0001664)	5.01E-03	*GNAZ*; *GNA14*; *GNA15*; *GNA11*; *GNAQ*
G-protein-coupled purinergic nucleotide receptor activity (GO:0045028)	1.00E-01	*P2RY12*
G-protein-coupled receptor activity (GO:0004930)	1.49E-01	*P2RY12*; *GNRHR2*; *HTR1E*
Unfavorable		
G-protein-coupled serotonin receptor activity (GO:0004993)	1.69E-01	*HTR1A*
G-protein-coupled receptor activity (GO:0004930)	1.73E-01	*CASR*; *NMUR2*; *NMUR1*
G-protein-coupled amine receptor activity (GO:0008227)	1.86E-01	*HTR1A*
G-protein-coupled peptide receptor activity (GO:0008528)	3.67E-01	*NMUR1*

**TABLE 3:** PathFX network proteins with high (“favorable,” high phenoscore), or low (“unfavorable,” low phenoscore) coefficients from linear regression.

Top 10 genes and regression coefficients (“favorable”)		
Gene symbol	Gene name	Regression coefficient
*CYP11B2*	Cytochrome P450 family 11, subfamily B, member 2	0.077
*GABRA1*	Gamma-aminobutyric acid type a receptor subunit alpha 1	0.076
*TTR*	Modeling transthyretin	0.066
*AKR1C3*	Aldo-keto reductase family member C3	0.060
*DERL1*	Derlin 1	0.048
*ADRA2A*	Adrenoceptor alpha 2A	0.042
*UGT1A3*	UDP-glucuronosyltransferase 1A3	0.037
*SLC22A3*	Solute carrier family 22, member 3	0.036
*ADRA2B*	Alpha-2B adrenergic receptor	0.033
*ADRA2C*	Adrenoceptor alpha 2C	0.033
Bottom 10 genes and regression coefficients (“unfavorable”)		
Gene symbol	Gene name	Regression coefficient
*DRD4*	Dopamine D4 receptor	−0.170
*KLK3*	KLK3 kallikrein-related peptidase 3	−0.083
*SLC22A4*	Solute carrier family 22, member 4	−0.071
*POR*	Cytochrome p450 oxidoreductase	−0.058
*SLC22A5*	Solute carrier family 22, member 5	−0.051
*SLCO1A2*	Solute carrier organic anion transporter family member 1A2	−0.051
*ABCC6*	ATP-binding cassette subfamily C, member 6	−0.049
*PDE4B*	Phosphodiesterase 4B	−0.036
*SLCO2B1*	Solute carrier organic anion transporter family member 2B1	−0.034
*AHR*	Aryl hydrocarbon receptor	−0.033
*CACNA1I*	Calcium voltage-gated channel subunit alpha 1 I	−0.033

**TABLE 4:** Select GO terms associated with favorable/unfavorable network proteins using zebrafish phenoscores.

Term	Adjusted P-value	Gene
Favorable		
Oxidoreductase activity, acting on paired donors, with incorporation or reduction of molecular oxygen, NAD(P)H as one donor, and incorporation of one atom of oxygen (GO:0016709)	3.59E-04	*CYP27A1*; *CYP27B1*; *AKR1C3*; *FMO1*
Voltage-gated sodium channel activity (GO:0005248)	2.84E-03	*SCN10A*; *SCN5A*; *SCN2A*
Epinephrine binding (GO:0051379)	5.02E-03	*ADRA2C*; *ADRA2B*
Voltage-gated calcium channel activity (GO:0005248)	1.67E-02	*SCN10A*; *SCN5A*; *SCN2A*
Oxidoreductase activity, acting on NAD(P)H, quinone, or similar compound as acceptor (GO: 0016655)	2.24E-02	*NQO1*; *AKR1C3*
Adrenergic receptor binding (GO:0031690)	2.24E-02	*DLG4*; *ADRA2C*
Hexose transmembrane transporter activity (GO:0015149)	2.27E-02	*SLC2A2*; *PPBP*
Oxidoreductase activity, acting on the aldehyde or oxo group of donors, NAD or NADP as acceptor (GO:0016620)	2.91E-02	*ALDH2*; *AKR1C3*
Steroid hydroxylase activity (GO:0008395)	3.52E-02	*CYP27A1*; *CYP11B2*
Glucose transmembrane transporter activity (GO:0005355)	3.67E-02	*SLC2A2*; *PPBP*
Unfavorable		
G-protein-coupled serotonin receptor activity (GO:0004993)	8.38E-10	*HTR6*; *HTR7*; *HTR1A*; *HTR2B*; *HTR1B*; *HTR2C*; *DRD4*
Serotonin receptor activity (GO:0099589)	2.32E-09	*HTR6*; *HTR7*; *HTR2B*; *HTR1A*; *HTR2C*; *HTR1B*; *DRD4*
G-protein-coupled amine receptor activity (GO:0008227)	2.77E-09	*HTR6*; *HTR7*; *HTR2B*; *HTR1A*; *HTR2C*; *HTR1B*; *DRD4*
3′,5′-Cyclic-nucleotide phosphodiesterase activity (GO:0004114)	1.12E-05	*PDE4D*; *PDE4C*; *PDE4B*; *PDE4A*
cAMP binding (GO:0030552)	3.10E-04	*PDE4D*; *PDE4B*; *PDE4A*
Transmitter-gated monoatomic ion channel activity (GO:0022824)	5.88E-04	*GRIA2*; *GABRA6*; *GRIK2*; *GRIA4*
Postsynaptic neurotransmitter receptor activity (GO:0098960)	1.06E-03	*CHRNA7*; *DRD4*; *DRD5*
Neurotransmitter receptor activity involved in regulation of postsynaptic membrane potential (GO: 0099529)	1.28E-03	*GRIA2*; *GRIK2*; *GRIA4*
Glutamate receptor activity (GO:0008066)	1.84E-02	*GRIA2*; *GRIA4*
G-protein-coupled receptor activity (GO:0004930)	4.12E-02	*GABBR1*; *GNRHR2*; *PTGDR2*; *DRD5*

**TABLE 5:** PathFX network proteins with high (“favorable,” increased clinical efficacy) or low (“unfavorable,” relatively lower clinical efficacy) coefficients from linear regression.

Top 10 genes and regression coefficients (“favorable”)		
Gene symbol	Gene name	Regression coefficient
ABCG2	ATP-binding cassette subfamily G, member 2	0.193
SLC6A4	Solute carrier family 6, member 4	0.152
CYP1A2	Cytochrome P450 family 1, subfamily a member 2	0.063
SLC6A2	Solute carrier family 6, member 2	0.056
ABCB1	ATP-binding cassette subfamily B, member 1	0.050
CYP2C19	Cytochrome P450 family 2 subfamily C, member 19	0.045
TAS2R60	Taste receptor type 2, member 60	0.038
OXGR1	Oxoglutarate receptor 1	0.038
XCL2	X-C motif chemokine ligand 2	0.038
HTR1D	5-Hydroxytryptamine receptor 1D	0.032
Bottom 10 genes and regression coefficients (“unfavorable”)		
Gene symbol	Gene name	Regression coefficient
CYP2D6	Cytochrome P450 family 2, subfamily D, member 6	−0.179
CYP3A4	Cytochrome P450 family 3, subfamily a, member 4	−0.052
CYP3A5	Cytochrome P450 family 3, subfamily a, member 5	−0.034
HTR2A	5-Hydroxytryptamine receptor 2A	−0.031
GSTP1	Glutathione S-transferase pi 1	−0.029
SLC6A3	Solute carrier family 6, member 3	−0.029
ADRA1B	Adrenoceptor alpha 1B	−0.026
TBXA2R	Thromboxane A2 receptor	−0.021
ADRA1A	Adrenoceptor alpha 1A	−0.021
ITGB5	Integrin beta-5	−0.019

**TABLE 6:** Select GO terms associated with favorable/unfavorable network proteins using clinical efficacy data.

Term	Adjusted P-value	Gene
Favorable		
G-protein-coupled serotonin receptor activity (GO:0004993)	9.05E-08	*HTR1E*; *HTR1F*; *HTR1D*; *HTR5A*; *DRD4*
Serotonin receptor activity (GO:0099589)	1.44E-07	*HTR1E*; *HTR1F*; *HTR1D*; *HTR5A*; *DRD4*
G-protein-coupled amine receptor activity (GO:0008227)	1.44E-07	*HTR1E*; *HTR1F*; *HTR1D*; *HTR5A*; *DRD4*
Epinephrine binding (GO:0051379)	2.77E-06	*ADRA2C*; *ADRA2B*; *DRD4*
Steroid hydroxylase activity (GO:0008395)	3.92E-06	*CYP2C9*; *CYP1A2*; *CYP1A1*; *CYP2C19*
G-protein-coupled opioid receptor activity (GO:0004985)	4.40E-04	*OPRD1*; *OPRM1*
Neurotrophin binding (GO:0043121)	8.50E-04	*NTRK1*; *NTRK2*
G-protein-coupled receptor activity (GO:0004930)	1.60E-03	*TAS2R60*; *OPRD1*; *GNAT2*; *HTR1E*; *OPRM1*
G-protein-coupled receptor binding (GO:0001664)	1.69E-03	*GNAO1*; *GNAZ*; *NPB*; *GNAT2*
Protein tyrosine kinase binding (GO:1990782)	4.84E-03	*SHC2*; *SHC3*; *CRK*
Neuropeptide binding (GO:0042923)	9.20E-03	*OPRD1*; *OPRM1*
Unfavorable		
G-protein-coupled serotonin receptor activity (GO:0004993)	4.77E-03	*HTR1A*; *HTR2A*
Steroid hydroxylase activity (GO:0008395)	4.77E-03	*CYP3A4*; *CYP3A5*
Serotonin receptor activity (GO:0099589)	5.07E-03	*HTR1A*; *HTR2A*
G-protein-coupled amine receptor activity (GO:0008227)	5.07E-03	*HTR1A*; *HTR2A*
GTPase regulator activity (GO:0030695)	1.52E-02	*TIAM1*; *TBXA2R*; *GCGR*; *ADRB1*
G-protein-coupled peptide receptor activity (GO:0008528)	2.94E-02	*NPSR1*; *GCGR*

**TABLE 7:** Selected shared favorable and unfavorable genes across all screens. Note: negative coefficients were considered “favorable” in the *in silico* screen only.

Gene	*in silico*	Zebrafish	Clinical	Average
Favorable				
*ABCG2*	−0.0228	0.0012	0.2249	0.0830
*SLC6A4*	−0.0043	0.0128	0.1668	0.0613
*GNAZ*	−0.0906	0.0276	0.0173	0.0452
*ADRA2A*	−0.0761	0.0420	0.0173	0.0451
*HTR1D*	−0.0745	0.0002	0.0329	0.0359
*HTR1E*	−0.0745	0.0002	0.0329	0.0359
*HTR1F*	−0.0745	0.0002	0.0329	0.0359
*HTR5A*	−0.0745	0.0002	0.0329	0.0359
*ADORA3*	−0.0900	0.0144	0.0005	0.0350
*ADRA2B*	−0.0529	0.0334	0.0173	0.0345
*ADRA2C*	−0.0529	0.0334	0.0173	0.0345
*DERL1*	−0.0098	0.0484	0.0134	0.0239
*CYP1A1*	−0.0482	0.0111	0.0110	0.0235
*ANXA1*	−0.0513	0.0070	0.0105	0.0229
*ABCG2*	−0.0228	0.0012	0.2249	0.0830
Unfavorable				
*PDE4B*	0.1172	−0.0364	−0.0191	0.0576
*HTR1A*	0.1355	−0.0143	−0.0105	0.0534
*SLCO2B1*	0.0651	−0.0341	−0.0004	0.0332
*GABBR1*	0.0303	−0.0187	−0.0191	0.0227
*HTR2A*	0.0148	−0.0019	−0.0362	0.0176
*TAC1*	0.0216	−0.0013	−0.0026	0.0085
*F2*	0.0155	−0.0023	−0.0026	0.0068
*GRM5*	0.0049	−0.0013	0.0110	0.0057
*ADRA1D*	0.0049	−0.0013	−0.0052	0.0038
*NMBR*	0.0049	−0.0013	0.0042	0.0035
*GPR68*	0.0049	−0.0013	−0.0042	0.0035
*P2RY10*	0.0049	−0.0013	−0.0042	0.0035
*GRPR*	0.0049	−0.0013	−0.0040	0.0034
*PROKR2*	0.0049	−0.0013	−0.0027	0.0030
*PDE4B*	0.1172	−0.0364	−0.0191	0.0576

**TABLE 8:** Selected shared and significant GO terms across all screens.

Term	Adj P-value *in silico*	Genes *in silico*	Adj P-value *in vivo*	Genes *in vivo*	Adj P-value clinical	Genes clinical
Favorable						
Monoamine transmembrane transporter activity (GO:0008504)	1.26E-05	*SLC22A3*; *SLC22A2*; *SLC18A2*; *SLC6A3*	2.14E-05	*SLC22A3*; *SLC6A2*; *SLC6A3*; *SLC6A4*	1.69E-03	*SLC6A2*; *SLC6A4*
Sodium:chloride symporter activity (GO:0015378)	1.41E-02	*SLC6A3*; *SLC18A2*	1.07E-03	*SLC6A2*; *SLC6A3*; *SLC6A4*	2.26E-03	*SLC6A2*; *SLC6A4*
Steroid hydroxylase activity (GO: 0008395)	2.63E-02	*CYP11A1*; *CYP1A1*	3.52E-02	*CYP27A1*; *CYP11B2*	3.92E-06	*CYP2C9*; *CYP1A2*; *CYP1A1*; *CYP2C19*
G-protein-coupled receptor binding (GO:0001664)	5.01E-03	*GNAZ*; *GNA14*; *GNA15*; *GNA11*; *GNAQ*	5.33E-01	*GNAZ*	1.69E-03	*GNAO1*; *GNAZ*; *NPB*; *GNAT2*
Epinephrine binding (GO: 0051379)	7.90E-02	*ADRB3*	5.02E-03	*ADRA2C*; *ADRA2B*	2.77E-06	*ADRA2C*; *ADRA2B*; *DRD4*
Unfavorable						
Serotonin receptor activity (GO: 0099589)	3.93E-02	*HTR1A*; *HTR3A*	2.32E-09	*HTR6*; *HTR7*; *HTR2B*; *HTR1A*; *HTR2C*; *HTR1B*; *DRD4*	5.07E-03	*HTR1A*; *HTR2A*
3′,5′-Cyclic-AMP phosphodiesterase activity (GO: 0004115)	3.21E-05	*PDE4D*; *PDE4C*; *PDE4B*; *PDE4A*	1.12E-05	*PDE4D*; *PDE4C*; *PDE4B*; *PDE4A*	5.29E-02	*PDE4B*
3′,5′-Cyclic-nucleotide phosphodiesterase activity (GO: 0004114)	4.04E-05	*PDE4D*; *PDE4C*; *PDE4B*; *PDE4A*	2.34E-05	*PDE4D*; *PDE4C*; *PDE4B*; *PDE4A*	5.29E-02	*PDE4B*
cAMP binding (GO:0030552)	3.63E-04	*PDE4D*; *PDE4B*; *PDE4A*	3.10E-04	*PDE4D*; *PDE4B*; *PDE4A*	5.24E-02	*PDE4B*
Cyclic nucleotide binding (GO: 0030551)	1.57E-03	*PDE4D*; *PDE4B*; *PDE4A*	1.28E-03	*PDE4D*; *PDE4B*; *PDE4A*	6.00E-02	*PDE4B*
G-protein-coupled serotonin receptor activity (GO:0004993)	1.69E-01	*HTR1A*	8.38E-10	*HTR6*; *HTR7*; *HTR1A*; *HTR2B*; *HTR1B*; *HTR2C*; *DRD4*	4.77E-03	*HTR1A*; *HTR2A*
G-protein-coupled amine receptor activity (GO:0008227)	1.86E-01	*HTR1A*	2.77E-09	*HTR6*; *HTR7*; *HTR2B*; *HTR1A*; *HTR2C*; *HTR1B*; *DRD4*	5.07E-03	*HTR1A*; *HTR2A*

## Data Availability

Code and data related to this project are included in https://github.com/jenwilson521/trans_psych_net. We included all results from PathFX analysis, regression analysis, and GO enrichment. We have not provided the raw data from Gravina et al. (2022), McCarroll et al. (2019), or Bruni et al. (2016) but have provided instructions for accessing the data and code for parsing the raw data once obtained from the original source.
